# Clinical Teaching Fellow programmes as a strategy to support the delivery of NHS workforce priorities: a scoping review protocol

**DOI:** 10.1136/bmjopen-2025-113949

**Published:** 2026-04-24

**Authors:** Nicola Jones, Zilley Khan, Riikka Hofmann

**Affiliations:** 1 Royal Papworth Hospital NHS Foundation Trust, Cambridge, UK; 2 Faculty of Education, University of Cambridge, Cambridge, UK

**Keywords:** MEDICAL EDUCATION & TRAINING, Health Workforce, Health Services

## Abstract

**Introduction:**

The National Health Service (NHS) faces mounting pressure from an ageing population and the backlog of care following the COVID-19 pandemic. The NHS Long Term Workforce Plan sets out a strategic framework to address these pressures through three priorities: train, retain and reform. The plan outlines a range of measures, including the doubling of medical school places over the next decade. Realisation of these ambitions is constrained by limited training capacity, as existing educators face significant pressures due to clinical demands. Clinical Teaching Fellow (CTF) programmes provide resident doctors with protected time for education and may help expand capacity and alleviate workforce pressures on established educators. Despite their rapid growth, CTF programmes remain under-described, and their contribution to NHS workforce priorities has not been systematically examined. To address this gap, this scoping review will map published and unpublished evidence on UK-based CTF programmes, engaging knowledge users to ensure findings are relevant to practice and workforce priorities.

**Methods and analysis:**

The review will follow the Joanna Briggs Institute methodology for scoping reviews and reported in line with the Preferred Reporting Items for Systematic Reviews and Meta-Analyses (PRISMA) extension for Scoping Reviews. Evidence will be identified from academic databases (British Education Index, EMBASE, ERIC, MEDLINE, Scopus, Web of Science) and grey literature sources (Google Scholar, NHS and medical school websites, deanery pages and job-advertising platforms). Two reviewers will independently screen and extract data using a piloted form, with discrepancies resolved by discussion or a third reviewer. Extracted data will undergo descriptive analysis and narrative synthesis, guided by a Theory of Change framework to identify how CTF programme inputs, activities and outcomes relate to NHS workforce priorities. Knowledge users will be engaged throughout the review to refine research questions, inform source selection, interpret findings, and shape dissemination.

**Ethics and dissemination:**

Ethical approval has been granted. All participants will provide informed consent. Participant contributions will be pseudonymised, and data will be managed in accordance with UK data protection legislation. Dissemination will be informed by knowledge users to ensure that findings on CTF programmes, including reported outcomes and identified gaps, are shared with those involved in delivering or supporting CTF programmes and with NHS stakeholders responsible for workforce priorities in training, retention and reform.

**STRENGTHS AND LIMITATIONS OF THIS STUDY**

- A rigorous methodological approach will ensure a systematic and transparent review process.
- Comprehensive searches of academic databases and grey literature will reduce publication bias and capture practice-based, contextually rich insights.
- Active engagement of knowledge users will enhance the relevance and applicability of findings.
- Adopt a theory-informed approach to examine *how and why* Clinical Teaching Fellow (CTF) programmes may generate outcomes, including attention to the mechanisms through which they contribute to clinical learning environments and workforce development.
- Focusing on UK programmes may limit international generalisability but will ensure direct relevance to NHS workforce priorities.

## Introduction

### Pressure on the National Health Service: a growing problem

 The National Health Service (NHS) continues to experience significant and sustained pressure from a growing and ageing population, together with the backlog of elective procedures due to the COVID-19 pandemic.^1 2^ While restoring elective services is a national priority, persistent workforce shortages present a major constraint.^3 4^

These pressures affect both patients and healthcare professionals. Record numbers of patients await elective treatment, many exceeding target waiting times, and a significant proportion of emergency admissions experience prolonged delays.^5 6^ These challenges contribute to worsening health outcomes^7^ and declining public satisfaction with the NHS.^8^

Healthcare professionals are also affected. Findings from the NHS Staff Survey highlight widespread dissatisfaction, with inadequate staffing contributing to stress, burnout and poorer health and well-being, as well as high rates of sickness and growing intentions to leave the NHS.^9^ Collectively, these factors intensify workforce shortages^10^ further eroding staff well-being and compromising the delivery of patient care.

### Reducing pressure: the NHS workforce plan

The NHS Long Term Workforce Plan outlines a strategic response to the escalating workforce crisis, structured around three priorities: training, retention and reform.^3^ A central measure is the commitment to double the number of medical school places to 15 000 per year by 2031–2032, with the aim of expanding the medical workforce by 60 000 doctors by 2036–2037. Realising this ambition is contingent on the availability of sufficient skilled educators. However, concerns have been raised that the scale of expansion could place unsustainable demands on educator capacity.^11^ These concerns are supported by evidence from the General Medical Council’s (GMC) 2024 National Training Survey which found that half of trainers are at moderate or high risk of burnout, and almost 30% are unable to use their allocated training time because of clinical pressures.^12^ In recognition of these challenges, NHS England has introduced the Educator Workforce Strategy, which outlines actions to strengthen educator capacity, protect time for training, and support workforce expansion.^13^ In response, national policy has emphasised strengthening the clinical educator workforce as a core enabler of workforce expansion.^14^ In addition, recent analyses highlight financial and infrastructural challenges associated with expanding medical training capacity, including funding constraints, limitations in clinical placement capacity and the need for additional facilities and supervision to support larger cohorts of learners.^15 16^

### Clinical Teaching Fellow programmes: a potential solution

Clinical Teaching Fellow (CTF) programmes represent one emerging approach to strengthening the clinical educator workforce needed to deliver the NHS Long Term Workforce Plan. CTF posts are undertaken by resident doctors who take time out of postgraduate training and are given protected time to deliver teaching, support learners in clinical environments and develop their skills as educators.^17^

Since their introduction in the late 1990s, CTF posts have expanded considerably and now account for approximately 10% of all advertised medical jobs in the UK.^18^ CTF posts may provide additional educator capacity and thereby support the planned expansion of medical school places, enabling the training of more doctors and contributing to the realisation of the NHS Long Term Workforce Plan.

Beyond expanding training capacity, CTF programmes may also support the wider priorities of retention and reform set out in the NHS Long Term Workforce Plan. By reducing pressure on consultants and other senior clinicians, CTF roles can help support a more sustainable balance between clinical service and educational responsibilities, conditions associated with improved retention.^19^ For resident doctors undertaking CTF roles, the reduction in clinical intensity and on-call commitments may enhance well-being and mitigate burnout, also recognised as a key determinant of retention in postgraduate medical training.^20^ In parallel, CTF programmes may advance educational reform through near-peer teaching,^21^ with fellows drawing on their recent transition into clinical practice to strengthen learners’ preparedness for work and foster innovation in the design and delivery of teaching and supervision.^19^

CTF programmes also align with national efforts to strengthen the clinical educator workforce,^13^ which include increasing capacity, supporting educator development and establishing clearer educational career pathways to facilitate training expansion, improve clinical learning environments and enhance learner experience. Taken together, CTF programmes may represent a key intervention through which national workforce priorities for training, retention and reform can be delivered in practice.

### The knowledge gap

Despite their rapid expansion, CTF programmes in the UK remain under-described in the peer-reviewed literature, with most studies limited to descriptive accounts or small-scale evaluations.^22 23^ Their role in supporting NHS workforce priorities has not been systematically examined.^3^ Emerging largely from local practice rather than evidence-informed policy, these programmes lack comprehensive evaluation. Significant gaps remain in understanding which inputs and activities contribute to their intended outcomes, and the mechanisms through which they generate impact across diverse contextual factors, reflecting broader challenges in evaluating complex interventions.^24^ While existing workplace-based learning theories^25^ and concepts such as socio-cognitive congruence^26^ offer plausible pedagogical explanations for how CTFs may support learning, in clinical environments, these mechanisms have not yet been empirically examined across different programme contexts. There is also limited understanding of how CTF programmes compare with other educational or workforce interventions, and of their relative contributions to training, retention and reform.

This gap is particularly critical given the prevalence of CTF posts, which now represent a substantial proportion of advertised junior doctor roles and are associated with annual salaries ranging from £41 750 to £70 425 per post.^27^ While these roles enhance educator capacity,^19^ they concurrently reduce clinical capacity, raising important questions about their relative advantages, disadvantages and broader implications for workforce planning.^11^ Addressing this gap is essential to determine whether CTF programmes represent an effective strategy to support the NHS Long Term Workforce Plan and to assess their wider impact on education and healthcare systems.^3^ This aligns with national expectations that workforce interventions are evaluated to build an evidence base on what works, for whom, and under what conditions, to inform future recommendations for wider implementation in practice and policy.^28^

### Rationale for scoping review

A preliminary search of MEDLINE, the Cochrane Database of Systematic Reviews and Joanna Briggs Institute (JBI) Evidence Synthesis was conducted and no current or underway systematic or scoping reviews on this topic were identified. To address this gap, we propose a scoping review to systematically map the evidence on CTF programmes in the UK and evaluate their potential contribution as a strategy to support NHS workforce priorities through training, retention and reform. Although a previous scoping review has been conducted, it only identified 10 papers describing nine programmes, with just one originating from the UK, all were published more than a decade ago.^29^ As such, it provides limited insights into CTF programmes, their relevance to current NHS workforce priorities, or the mechanisms through which programme inputs and activities may bring about change.

This paucity of evidence likely reflects the review’s exclusive reliance on published sources and its lack of engagement with knowledge users—individuals directly involved in or with a vested interest in CTF programmes, medical education or workforce planning.^30 31^ Engaging knowledge users may help identify valuable unpublished sources, such as conference proceedings, institutional reports and job advertisements, with this grey literature collectively representing a critical repository of practice-based knowledge^32^ and helping to reduce publication bias while providing timely, contextually relevant insights.^33^

Nevertheless, scoping reviews provide a rigorous approach to addressing broad, exploratory questions where the literature is heterogenous and therefore not suited to the specific, comparative effectiveness questions required for systematic reviews.^30 34^ This pattern is consistent with other areas of medical and health professions education, where complex educational interventions vary widely and cannot be reduced to a single effectiveness outcome. For example, Hofmann *et al*^35^ found that in simulation-based medical education, substantial variation in programme models, learning outcome frameworks and evaluative approaches made a traditional systematic review inappropriate, necessitating a scoping review to map the field and clarify conceptual and practical patterns.

They can incorporate both published and unpublished literature^32^ and engage knowledge users to enhance comprehensiveness, relevance and applicability.^31 36^ When combined with approaches such as theory of change (ToC), they can also provide insights on the mechanisms through which complex interventions bring about change.^37 38^ For these reasons, we will undertake an updated scoping review to synthesise published and unpublished evidence on CTF programmes. The review will be co-created with knowledge users and guided by a ToC lens to enable a structured, theory-informed examination of how programme inputs, activities, outputs, outcomes and contextual factors may generate change, while also making explicit the underlying assumptions. This approach will address the existing knowledge gap and provide a stronger foundation for future evaluation of CTF programmes and their role in supporting NHS workforce priorities.

### Objectives

The primary objective of this scoping review is to examine if, how, and why CTF programmes may contribute to NHS workforce priorities relating to training, retention and service reform. The review will systematically identify, map and synthesise published and unpublished evidence on CTF programmes within the NHS. A ToC approach will be used to explore the mechanisms through which programme activities are understood to generate outcomes, and how these mechanisms are shaped by contextual conditions. The review will also identify conceptual, empirical and evaluative gaps to inform future research, evaluation and programme development.

### Research questions

To address the objective of this review, the following review questions have been developed using the Population–Concept–Context (PCC) framework recommended by the JBI. The PCC elements informing this review are described in detail in the eligibility criteria.

The overarching research question is:

What is known about CTF programmes in the UK and their potential contribution to NHS workforce priorities related to training, retention and service reform?

This is explored through the following supporting questions:

1. What are the stated aims and objectives of CTF programmes, and how are these positioned in relation to NHS workforce priorities?
2. What inputs, organisational structures and activities characterise CTF programmes, and how do these vary across settings?
3. What outputs and outcomes have been reported in relation to CTF programmes, and to what extent do these outcomes relate to training, retention and service reform?
4. Through what mechanisms are CTF programme activities understood to produce their outcomes, and how are these mechanisms shaped, enabled or constrained by contextual factors?
5. What conceptual, empirical and evaluative gaps exist in the current evidence base, and what are the implications for future research, evaluation and programme development?

## Methods

The review will be conducted in accordance with the JBI methodology^39^ and will report full search strategies and results in accordance with Preferred Reporting Items for Systematic reviews and Meta-Analyses extension for Scoping Reviews (PRISMA-ScR) and PRISMA Statement for Reporting Literature Searches in Systematic Reviews (PRISMA-S) guidelines.^40^ The completed PRISMA-ScR checklist is available as online supplemental file 1.

To ensure a rigorous, comprehensive and practice-informed approach, the review will be conducted by a multidisciplinary team with expertise in clinical practice, medical education, workforce development, learning theory and scoping review methodology. In addition, the review will engage knowledge users throughout its design and conduct, recognising their role in enhancing the relevance, applicability and impact of findings.^36^ By integrating diverse expertise across clinical, educational, methodological and practice domains, this scoping review team will be well positioned to conduct a robust synthesis of the existing evidence on CTF programmes.^31^ The review is expected to commence in March 2026 and to be completed within 6 months.

### Eligibility criteria

The eligibility criteria for selecting sources for this scoping review will be described using the PCC framework, in line with JBI guidance^39^ and selected to ensure alignment with the review’s objectives and research questions.

- Population: This review will include published and unpublished evidence on CTF programmes involving resident doctors at any stage of postgraduate training across all clinical specialties. Programmes involving non-medically qualified fellows will be excluded. Eligible CTF programmes must provide protected time for teaching and educational activities, either as a full-time education post or part-time in combination with clinical duties. Programmes without designated time for these activities will be excluded.
- Concept: This scoping review will examine CTF programmes through a ToC framework,^38^ mapping their inputs, activities, outputs and outcomes in relation to NHS priorities to train, retain and reform the medical workforce. It will synthesise published and unpublished evidence to clarify how CTF programmes contribute to these workforce priorities.
- Context: this review will focus on CTF programmes within NHS trusts and UK medical schools and postgraduate deaneries. Programmes based in private healthcare settings or outside the UK will be excluded to ensure relevance to NHS workforce priorities.

### Evidence sources

This review will include both published and unpublished sources to comprehensively map evidence on CTF programmes. Incorporating grey literature helps reduce publication bias and capture practice-based, contextually rich evidence not typically available in academic journals.^30 32 33^

- Published evidence: In line with guidance that educational interventions in clinical settings require searching across health, education and organisational sciences due to dispersed evidence coverage (Cochrane EPOC; JBI), searches will be conducted in British Education Index (educational policy, administration, evaluation), ERIC (pedagogy and educator development), MEDLINE (Ovid) and EMBASE (clinical and medical education within postgraduate training) and Scopus and Web of Science (interdisciplinary organisational and workforce research). A pilot search will be undertaken to assess relevance and unique yield, and databases contributing minimal additional value may be excluded to maintain efficiency and transparency.
- Unpublished evidence: Similarly, to capture current, practice-based insights not available in peer-reviewed literature, unpublished evidence will be sought from grey literature databases, Google and Google Scholar (in incognito mode) and webpages of NHS organisations, UK medical schools and postgraduate deaneries, where locally produced programme documents, internal evaluations and operational guidance are commonly shared. Job advertisement platforms (eg, *BMJ Careers*, *NHS Jobs*) will also be included as these are recognised as a valuable data source for workforce research and have been used successfully in previous scoping and narrative reviews to capture role structures and workforce development trends.^41 42^ A pilot phase will be used to assess relevance and refine sources accordingly.

### Search strategy

The search strategy will follow a three-stage approach to systematically identify published and unpublished sources on CTF programmes. The strategy will be developed in collaboration with an academic librarian to ensure methodological rigour and comprehensive coverage.^39 43^ A draft full electronic search strategy for selected databases is provided in online supplemental file 2.

- Stage 1: Preliminary search—A pilot search will be conducted in MEDLINE (Ovid) and Embase (Embase.com) using terms such as ‘Clinical Teaching Fellow’ OR ‘Medical Education Fellow’. Title and abstract text words, along with index terms from retrieved records, will inform the development of a comprehensive search strategy. No restrictions on language, publication date or publication type will be applied, in line with JBI guidance to maximise inclusivity.^39^
- Stage 2: Systematic search—The search strategy will then be refined and adapted for academic databases and grey literature sources. Pilot testing will be applied across both published and unpublished sources to assess feasibility, coverage and relevance before finalising the search strategy. No restrictions on publication date will be applied to academic databases and grey literature to ensure comprehensive evidence mapping. For webpages from NHS organisations, UK medical schools and postgraduate deaneries, only those publicly accessible at the time of the search and describing CTF programmes, recruitment or related workforce initiatives will be included, as archived or outdated pages may not reflect current programmes. For job advertisement platforms, only adverts from the past 12 months will be included, as these are time-sensitive sources that reflect current recruitment practices, role descriptions and workforce structures, including older adverts risks capturing posts that no longer represent current CTF programme design or NHS workforce priorities.
- Stage 3: Reference list and citation searching—Citation tracking will be conducted iteratively using backward and forward searching in Web of Science, Scopus and Google Scholar. This approach is consistent with recommended best practice for systematic searching^44^ and will draw on recent tools developed to enhance transparency and reproducibility.^45^ The search process will be iteratively refined to ensure comprehensive and systematic identification of relevant sources. Search results will first be exported to Mendeley for reference management and, where necessary, conversion into PDFs. References will then be imported into JBI SUMARI, where deduplication, study screening, source selection and data extraction will be conducted, facilitating a transparent and reproducible process.

### Source selection

The PCC eligibility criteria will be piloted on an initial subset of records to ensure clarity and consistency in decision-making. Piloting will continue until reviewers demonstrate convergence on both inclusion and exclusion decisions, indicating that the criteria are clear, discriminating and workable in practice.^39^ Two independent reviewers will screen all identified sources at the title and abstract level, with full texts retrieved for further assessment if deemed relevant.

Full-text screening will be conducted independently by the same two reviewers, applying the eligibility criteria to determine final inclusion. To ensure consistency and reduce bias, a calibration exercise will be conducted on a subset of 25 studies, with an inter-rater agreement threshold of ≥85%^39^ and Cohen’s kappa reported.^46^ Discrepancies will be resolved through discussion, and if consensus cannot be reached, a third reviewer will adjudicate.^31^

**Figure 1:**
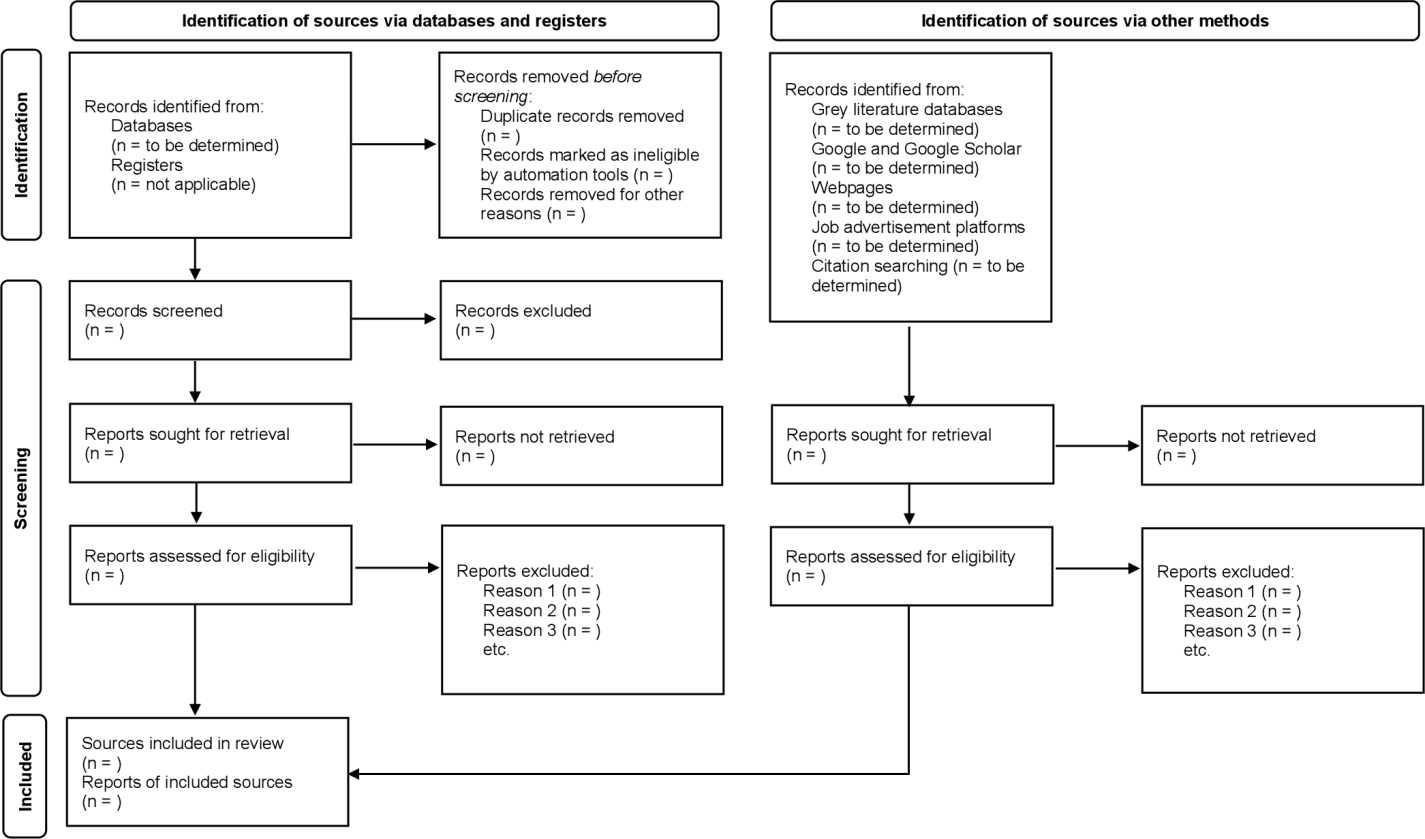
Preferred Reporting Items for Systematic reviews and Meta-Analyses extension for Scoping Reviews (PRISMA-ScR) flow diagram of the study selection process. Flow diagram illustrating the identification, screening, eligibility assessment and inclusion of published and unpublished sources identified through database searches and additional methods.

Excluded sources will be documented, with reasons for exclusion reported at the full-text screening stage. The study selection process will be presented in a PRISMA-ScR flow diagram (figure 1), ensuring transparent reporting of the identification, screening, eligibility and inclusion of sources.^40^

### Data extraction

Key components of CTF programmes will be extracted, including inputs, activities, outputs, outcomes and contextual factors. These components will be structured to align with the ToC framework, which will guide the mapping of how CTF programme activities and contexts may contribute to reported outputs and outcomes. Particular attention will be given to their potential contribution to NHS workforce priorities to train, retain and reform the medical workforce.

A data extraction form will be developed to capture this information, in alignment with the PCC framework and best practice for scoping reviews.^31^ The form will be piloted on 10 sources to assess feasibility, consistency and reliability in capturing relevant data.^39^ Inter-rater reliability will be assessed during this pilot, with a target of ≥85% agreement between reviewers, and Cohen’s kappa reported to provide a chance-adjusted measure of consistency.^46^ The form will be refined iteratively based on pilot findings^30 31^ and any modifications will be documented in the final review. The finalised data extraction tool will be presented in the scoping review to ensure transparency and reproducibility, in accordance with PRISMA-ScR reporting standards.^40^

### Data analysis and presentation

Extracted data will undergo descriptive analysis and narrative synthesis. Descriptive analysis will provide a structured overview of the included sources, summarising publication status, source type, origin, year and key characteristics. Where relevant, numerical summaries (counts and percentages) will be used to illustrate trends. Because scoping reviews do not appraise study quality, no formal risk of bias assessment will be undertaken.

Narrative synthesis will be guided by a ToC framework, mapping extracted data on inputs, activities, outputs, outcomes, impacts and contextual factors of CTF programmes, with particular attention to their contribution to training, retention, workforce reform and long-term NHS priorities. This approach will also allow exploration of the pedagogical mechanisms through which CTFs may support learning and integration in clinical environments, including those proposed in workplace-based learning theory and socio-cognitive congruence. In addition, Hofmann’s ‘difference-in-similarity’ approach, a qualitative method that involves first identifying a shared pattern within the dataset and then systematically examining the variation within that similarity to generate new theoretical insights.^47^ Findings will be presented in tables, supported by narrative explanation, and visualised using ToC-based diagrams and charts to illustrate relationships between programme components and intended impacts.

Given the iterative nature of scoping reviews, the analytical approach and presentation methods may be refined as insights emerge; any refinements will be transparently documented to maintain methodological rigour. Thematic patterns and research gaps will be identified to inform future research, policy and practice. The final report will follow the JBI Manual for Evidence Synthesis and PRISMA-ScR reporting guidelines.^39 40^

### Knowledge user engagement

Knowledge user engagement will occur throughout the review to refine the aims, research questions and search strategy; shape eligibility criteria and source selection; and discuss findings, implications and dissemination strategies, ensuring inclusivity and relevance.^36^

In the context of CTF programmes, there is no standardised definition of knowledge users. For this scoping review, knowledge users are defined as ‘individuals directly involved in or having an interest in CTF programmes, medical education or workforce planning’. This definition ensures the representation of a broad range of professional perspectives, supporting equity, diversity and inclusion in the research process.^48 49^

### Knowledge users

- Medical students and junior doctors taught by CTFs—Provide insights into the impact of CTFs on clinical training, particularly across diverse learner experiences.
- Current or recent CTFs (within 5 years**)**—Share experiences of the role, training and career progression, reflecting on any structural barriers or opportunities in the role.
- Consultants working alongside or supervising CTFs—Offer perspectives on how CTFs contribute to clinical education and whether these roles support inclusive and sustainable workforce development.
- Medical School Deans and Directors of Medical Education—Provide strategic insights into workforce and educational impact, with attention to policies promoting inclusivity in faculty development.
- Postgraduate Deans—Offer expertise on postgraduate training and workforce transitions, including considerations for diverse career pathways.
- Academy of Medical Royal Colleges Workforce Committee and NHS England Medical Workforce Advisors—Contribute policy perspectives on workforce planning, particularly in relation to equality, diversity, and inclusion (EDI).
- GMC Members—Provide regulatory insights on professional standards and medical education, ensuring alignment with EDI principles in training and assessment.

### Recruitment strategy

A multi-modal recruitment strategy will be used to maximise participation and ensure diverse representation.^50^ This approach is supported by evidence demonstrating that multi-modal recruitment enhances engagement across diverse professional groups in clinical and educational settings.^51^

- Postal distribution—Flyers will be sent to medical schools, NHS trusts, postgraduate deaneries, medical education societies, royal colleges, NHS England and the GMC, requesting targeted outreach to underrepresented groups.
- Group email—Invitations will be distributed via institutional networks, with explicit encouragement for participation from knowledge users across diverse professional backgrounds.
- Personal email—Direct invitations will be sent to the researcher’s professional network and corresponding authors of relevant studies, ensuring representation from a broad demographic.
- Social media—Targeted announcements will be posted on Twitter, Facebook, LinkedIn and Google+, using inclusive messaging to encourage engagement from underrepresented communities.
- Snowball recruitmentExisting knowledge users will be encouraged to share invitations with colleagues, particularly those from diverse professional and demographic backgrounds.

### Engagement through an online forum

Engagement will take place through a secure moderated online discussion forum hosted within Microsoft Teams. Online forums have been recognised as effective, flexible platforms for participatory engagement in health research, offering accessibility and inclusivity for diverse stakeholders.^52 53^ Knowledge users will register and receive a unique identification and initial password. To maintain anonymity, they will be asked to use a pseudonym. The forum will allow users to respond to posts, initiate discussions and provide structured feedback at key review stages, consistent with guidance on stakeholder engagement in evidence syntheses.^36^

The forum will be monitored twice weekly, with feedback provided on how contributions inform the review. An impact log will record engagement activities and their influence, reported using the Guidance for Reporting Involvement of Patients and the Public short form (GRIPP2) checklist to ensure transparency and methodological rigour.^54^ Participants will receive recognition and acknowledgement in resulting publications. This structured and inclusive approach is designed to support meaningful knowledge user involvement and promote relevance to NHS workforce priorities and EDI principles.^48 49^

### Patient and public involvement

Patient and public involvement (PPI) will be embedded throughout the review to ensure patient perspectives inform its design, conduct and dissemination.^55^ The PPI lead from the affiliated NHS organisation will work with a diverse group of patient advisers to contribute at key stages, including question refinement, data extraction, interpretation and dissemination. Their input will help ensure that EDI^56^ are considered and that outcomes reflect what patients and the public view as most meaningful.^57^

Meetings will be held in face-to-face, online or hybrid formats to support accessibility. Reimbursement will follow National Institute for Health and Care Research (NIHR) Payment Guidance for PPI. All activities will align with the UK Standards for Public Involvement^58^ and an activity log maintained to record contributions and impact. Engagement will be reported using the GRIPP2 checklist to ensure transparency and consistency with national standards.^54^

### Ethics and dissemination

Ethical approval has been granted by the Faculty of Education, University of Cambridge (reference: UoC-EDU-121224-nlj-01E) for the knowledge user engagement activities involving human participants described in this study. Informed consent will be obtained from all participants, who may withdraw at any time without disadvantage. Participant contributions will be pseudonymised, and any institutional or personal identifiers within public sources will be de-identified before data extraction and reporting, in line with UK data protection legislation and General Data Protection Regulation (GDPR).^59 60^ Data handling and reporting will follow the JBI Manual for Evidence Synthesis and PRISMA-ScR guidelines.^39 40^ The dissemination strategy will be co-created with knowledge users to ensure findings are accessible and relevant to those involved in CTF programmes and NHS workforce priorities for training, retention and reform.

## Supplementary material

10.1136/bmjopen-2025-113949 online supplemental file 1

10.1136/bmjopen-2025-113949 online supplemental file 2

## Data Availability

No data are available.
