# Supplementary material for: Clinical Teaching Fellow programmes as a strategy to support the delivery of NHS workforce priorities: a scoping review protocol
Source: BMJ Open. 2026 Apr 24;16(4):e113949. doi: 10.1136/bmjopen-2025-113949 (PMC13110527; doi:10.1136/bmjopen-2025-113949)
Supplement: online supplemental file 2 [file bmjopen-16-4-s002.docx]

The following draft electronic search strategies were developed for this scoping review and are presented to support transparency and reproducibility. These strategies will be refined iteratively during the review process in consultation with an academic librarian. The searches below were run on 1 October 2025. Differences in displayed date ranges reflect the database platform coverage statements and update conventions.

**Embase <1974 to 2025 September 29>**

1 exp *united kingdom/ or exp *great britain/ or exp *northern ireland/ or exp *England/ or exp *Wales/ or exp *Scotland/ or exp *Ireland/ 52422

2 (united kingdom or UK or Britain or British or England or English or Wales or Welsh or Scotland or Scottish or Ireland or Irish).ti,ab,kw,in. 4345245

3 1 or 2 4348674

4 ((teach* or education* or educator* or simulation*) adj3 fellow*).ti,ab,kw. 3000

5 3 and 4 371

**Ovid MEDLINE(R) ALL <1946 to September 29, 2025>**

1 exp United Kingdom/ or exp Northern Ireland/ or exp England/ or exp Wales/ or exp Scotland/ or exp Ireland/ 426023

2 (united kingdom or UK or Britain or British or England or English or Wales or Welsh or Scotland or Scottish or Ireland or Irish).ti,ab,kw,in. 2753808

3 1 or 2 2948198

4 ((teach* or education* or educator* or simulation*) adj3 fellow*).ti,ab,kw. 1746

5 3 and 4 170

**HMIC Health Management Information Consortium <1979 to July 2025>**

1 exp United Kingdom/ or exp Great Britain/ or exp Northern Ireland/ or exp England/ or exp Wales/ or exp Scotland/ or exp Ireland/ 48840

2 (united kingdom or UK or Britain or British or England or English or Wales or Welsh or Scotland or Scottish or Ireland or Irish).ti,ab. 73285

3 1 or 2 99389

4 ((teach* or education* or educator* or simulation*) adj3 fellow*).ti,ab. 12

5 3 and 4 4
