# Supplementary material for: Clinical Teaching Fellow programmes as a strategy to support the delivery of NHS workforce priorities: a scoping review protocol
Source: BMJ Open. 2026 Apr 24;16(4):e113949. doi: 10.1136/bmjopen-2025-113949 (PMC13110527; doi:10.1136/bmjopen-2025-113949)
Supplement: Supplementary file 3 [file bmjopen-2025-113949.reviewer_comments.pdf]

PEER REVIEW HISTORY

BMJ Open publishes all reviews undertaken for accepted manuscripts. Reviewers are asked to complete a checklist review form and are provided with free text boxes to elaborate on their assessment. These free text comments are reproduced below.

ARTICLE DETAILS

Title (Provisional)

Clinical Teaching Fellow Programmes as a Strategy to Support the Delivery of NHS Workforce Priorities: A Scoping Review Protocol

Authors

Jones, Nicola; Khan , Zilley; Hofmann, Riikka

VERSION 1 - REVIEW

**Reviewer**	**1**
**Name**	**De Rezende, Helena**
**Affiliation**	**Bournemouth University, School of Nursing**
**Date**	**04-Mar-2026**
**COI**	**None**

Thank you for the opportunity to review this protocol. This is a very interesting and important topic, specifically at this time when the NHS is facing increasing pressures and challenges.

This is a very well-conducted scoping review protocol. I have minor considerations to add. Please see my comments below, and I hope this helps improve your protocol.

Abstract

Abstract should not include references.

Introduction

Reducing Pressure: The NHS Workforce Plan

Lines 38-39: In this part *Concerns have been raised that the scale of expansion could place unsustainable demands on educator capacity ...*

More information regarding the challenges of implementing medical schools should be added. You noted the staff's limitations. However, financial implications should also be discussed.

Please see 2 references to support this section:
https://www.sciencedirect.com/science/article/pii/S1470211825002416

https://pmc.ncbi.nlm.nih.gov/articles/PMC12116428/

Rationale for Scoping Review

Lines 20-21: as per JBI guidance, a preliminary search must be stated and other reviews conducted on this topic should be presented if is the case. Please see what JBI recommends being added (please modify accordingly):

A preliminary search of MEDLINE, the Cochrane Database of Systematic Reviews and JBI Evidence Synthesis was conducted and no current or underway systematic reviews or scoping reviews on the topic were identified. If there are any existing systematic reviews/scoping reviews, it should be specified how the proposed review will differ (in this part please add your statements about a previous review published, lines 24-26).

Research Questions:

Lines 29-30: Clearly state the review question or questions using the PCC framework (i.e. additional or sub-questions) that the review seeks to answer. The review question/s should clearly relate to the objective/s. The overarching question is a bit confusing; please follow the PCC framework as per JBI Guidance.

Methods

Line 57: Please also state that the review will be reported following Prisma ScR for scoping reviews (this was mentioned in the abstract but not in the main text). Move the sentence in stage 3 (see comment below) to here.

Stage 3: Reference list and citation searching

Please move this sentence *The review will report full search strategies and results in accordance with PRISMA-ScR and PRISMA-S guidelines (8), to* methods line 57 as above.

VERSION 1 - AUTHOR RESPONSE

Reviewer comments		
Abstract	Abstract should not include references	Thank you for noting this. We have removed the references from the abstract in the revised manuscript.
Introduction	Reducing Pressure: The NHS Workforce Plan Lines 38-39: In this part Concerns have been raised that the scale of expansion could place unsustainable demands on educator capacity ...<br>More information regarding the challenges of implementing medical schools should be added. You noted the staff's limitations. However, financial implications should also be discussed. Please see 2 references to support this section:<br>https://www.sciencedirect.com/science/article/pii/S1470211825002416<br>https://pmc.ncbi.nlm.nih.gov/articles/PMC12116428/	Thank you for highlighting this important point. We have revised this section to acknowledge the financial and infrastructural challenges associated with expanding medical training capacity, including funding constraints, clinical placement capacity, and infrastructure required to support larger cohorts of learners. The suggested references have been incorporated to support this addition.
Rationale for Scoping Review	Lines 20-21: as per JBI guidance, a preliminary search must be stated and other reviews conducted on this topic should be presented if is the case. Please see what JBI recommends being added (please modify accordingly): A preliminary search of MEDLINE, the Cochrane Database of Systematic Reviews and JBI Evidence Synthesis was conducted and no current or underway systematic reviews or scoping reviews on the topic were identified. If there are any existing systematic reviews/scoping reviews, it should be specified how the proposed review will differ (in this part please add your statements about a previous review published, lines 24-26)	Thank you for highlighting this important point. We have now added a statement describing the preliminary search conducted in MEDLINE, the Cochrane Database of Systematic Reviews, and JBI Evidence Synthesis, in line with JBI guidance. The manuscript already discussed the previous scoping review identified and clarifies how the proposed review differs, and this has been retained in the revised section.
Research Questions	Lines 29-30: Clearly state the review question or questions using the PCC framework (i.e.	Thank you for highlighting this important point. This

	additional or sub-questions) that the review seeks to answer. The review question/s should clearly relate to the objective/s. The overarching question is a bit confusing; please follow the PCC framework as per JBI Guidance.	comment prompted us to reflect on the clarity of the review questions in relation to the PCC framework. We have revised the Research Questions section to clarify that the review questions are informed by the Population–Concept–Context (PCC) framework recommended by the Joanna Briggs Institute, with the specific PCC elements described in the Eligibility Criteria section.
Methods	Line 57: Please also state that the review will be reported following Prisma ScR for scoping reviews (this was mentioned in the abstract but not in the main text). Move the sentence in stage 3 (see comment below) to here.	Thank you for noting this. We have now moved the sentence from stage 3 indicating that the review will be reported in line with the PRISMA extension for Scoping Reviews.
Stage 3: Reference list and citation searching	Please move this sentence The review will report full search strategies and results in accordance with PRISMA-ScR and PRISMA-S guidelines (8), to methods line 57 as above	Thank you for highlighting this. The sentence has now been moved to the Methods section as requested.
